# Predicting tumor deposits in rectal cancer: a combined deep learning model using T2-MR imaging and clinical features

**DOI:** 10.1186/s13244-023-01564-w

**Published:** 2023-12-20

**Authors:** Yumei Jin, Hongkun Yin, Huiling Zhang, Yewu Wang, Shengmei Liu, Ling Yang, Bin Song

**Affiliations:** 1 Department of Medical Imaging Center, Qujing First People’s Hospital, Qujing, 655000 Yunnan Province China; 2 Department of Radiology, West China Hospital of Sichuan University, Chengdu, 610041 Sichuan Province China; 3 Beijing Infervision Technology Co.Ltd, Beijing, China; 4 Department of Joint and Sports Medicine, Qujing First People’s Hospital, Qujing, 655000 Yunnan Province China; 5 Functional and Molecular Imaging Key Laboratory of Sichuan Province, West China Hospital of Sichuan University, Chengdu, 610041 Sichuan Province China; 6 Department of Radiology, Sanya People’s Hospital, Sanya, Hainan Province 572000 China

**Keywords:** Rectal cancer, Tumor deposits, Deep learning, Hybrid neural network

## Abstract

**Background:**

Tumor deposits (TDs) are associated with poor prognosis in rectal cancer (RC). This study aims to develop and validate a deep learning (DL) model incorporating T2-MR image and clinical factors for the preoperative prediction of TDs in RC patients.

**Methods and methods:**

A total of 327 RC patients with pathologically confirmed TDs status from January 2016 to December 2019 were retrospectively recruited, and the T2-MR images and clinical variables were collected. Patients were randomly split into a development dataset (*n* = 246) and an independent testing dataset (*n* = 81). A single-channel DL model, a multi-channel DL model, a hybrid DL model, and a clinical model were constructed. The performance of these predictive models was assessed by using receiver operating characteristics (ROC) analysis and decision curve analysis (DCA).

**Results:**

The areas under the curves (AUCs) of the clinical, single-DL, multi-DL, and hybrid-DL models were 0.734 (95% CI, 0.674–0.788), 0.710 (95% CI, 0.649–0.766), 0.767 (95% CI, 0.710–0.819), and 0.857 (95% CI, 0.807–0.898) in the development dataset. The AUC of the hybrid-DL model was significantly higher than the single-DL and multi-DL models (both *p* < 0.001) in the development dataset, and the single-DL model (*p* = 0.028) in the testing dataset. Decision curve analysis demonstrated the hybrid-DL model had higher net benefit than other models across the majority range of threshold probabilities.

**Conclusions:**

The proposed hybrid-DL model achieved good predictive efficacy and could be used to predict tumor deposits in rectal cancer.

**Critical relevance statement:**

The proposed hybrid-DL model achieved good predictive efficacy and could be used to predict tumor deposits in rectal cancer.

**Key points:**

• Preoperative non-invasive identification of TDs is of great clinical significance.

• The combined hybrid-DL model achieved good predictive efficacy and could be used to predict tumor deposits in rectal cancer.

• A preoperative nomogram provides gastroenterologist with an accurate and effective tool.

**Graphical Abstract:**

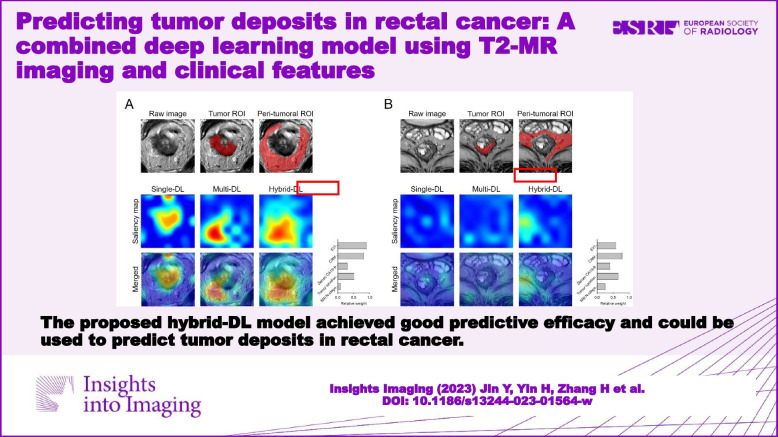

**Supplementary Information:**

The online version contains supplementary material available at 10.1186/s13244-023-01564-w.

## Introduction

As one of the most prevalent malignancies worldwide, colorectal cancer (CRC) is the leading cause of cancer-related death and a serious threat to people’s health and life quality [1]. According to the research results of International Agency for Research on Cancer(IARC), by 2040, the burden of colorectal cancer will increase to 3.2 million new cases per year (an increase of 63%) and 1.6 million deaths per year (an increase of 73%), increasing incidence rates are observed in younger adults and in countries that are undergoing economic transition [2]. Tumor deposits (TDs) are defined as discrete tumor foci located in the pericolonic or perirectal fat without histological evidence of residual lymph nodes or identifiable vascular or neural structures [3]. TDs are an independent risk factor for the prognosis of rectal cancel [4]. Tumors with positive TDs showed more aggressive biological behavior and are prone to local recurrence and distant metastases [5]. Patients with positive TDs show worse overall survival (OS) and disease-free survival (DFS) than patients with negative TDs [6]. TDs can ascend the N staging of tumor and are associated with patients’ treatment protocols, according to the 8th Tumor Node Metastasis (TNM) staging system of American Joint Committee on Cancer (AJCC), e.g., for any T stage patients, if TDs are positive, the patient will be classified as the stage of N1c and will be recommended for preoperative or postoperative neoadjuvant chemoradiotherapy (nCRT) instead of single surgical treatment, according to European Society of Medical Oncology (ESMO) consensus guidelines recommendation that nCRt is required in high-risk groups [7], and recent researches indeed demonstrated that patients with positive TD status and those who have undergone nCRT have a better survival outcome than those who have not undergone nCRT [8]. To sum up, the preoperative non-invasive identification of TDs is of great clinical significance, which plays a critical role in stage evaluation and treatment planning. However, the diagnosis of TDs primarily relies on postoperative pathological assessment, a method characterized by its inherent delay and invasiveness. The pursuit of non-invasive, precise, accurate preoperative diagnostic approaches is the focal point of our efforts.

The T2-weighted magnetic resonance imaging (T2WI-MRI) is the first-choice imaging modality for local staging in RC patients and can be used to evaluate the risk factors of rectal cancer, such as circumferential resection margin (CRM) status and the extramural vascular invasion (EMVI) [9], e.g., Yang et al. [10]. developed two nomograms through the integration of clinical risk factors, high-resolution magnetic resonance imaging (HRMRI) findings, and the research results demonstrated that the nomograms based on HRMRI and radiomics exhibited good predictive performance, with AUC values of 0.90 (95% confidence interval [CI] = 0.83–0.96) for LNM and 0.80 (95% CI = 0.69–0.92) for TDs, respectively. And Atre [11] undertook a retrospective analysis of the MRI imaging performance in 40 patients with pathologically confirmed TDs and malignant lymph nodes, and the research findings demonstrated that the morphology of lesions observed on MR imaging can serve as a valuable predictive factor for distinguishing TDs from positive lymph nodes (LNs) in patients with rectal cancer. Furthermore, when morphology information was combined with the MR texture parameter of skewness, diagnostic accuracy was significantly enhanced. However, the radiomics features defined by manually outlining the target region are pre-defined and highly dependent on the accurate segmentation of regions of interest (ROI); the inter-observer variability might challenge the reliability of the result [12]. In addition, the stability of texture features varies among different image acquisition and preprocessing, which could also limit its practical application [13].

In recent years, deep learning (DL), especially the convolutional neural network (CNN), has provided a new approach to medical image analysis and raised much attention in clinical practice. Compared with conventional machine learning, deep learning can automatically extract features from an image without the necessity of feature predefinition and is suitable for mining the most relevant feature representations [14–17]. TDs are scattered and distributed within the peritumoral adipose tissue, and our analysis focuses on the adipose tissue surrounding the tumor, for which deep learning methods are well-suited for the study of the imaging area. Moreover, there were researches about the application of DL models on MR imaging in predicting microsatellite instability and nCRT outcome [18, 19]; whether the DL algorithms could help to characterize the TDs status in RC patients has not been reported yet. So this study aims to construct a DL model incorporating both T2-MR images and clinical risk factors and assess its diagnostic accuracy for predicting TDs in RC patients.

## Methods

### Patient enrollment and study design

The study was conducted in accordance with the Declaration of Helsinki (as revised in 2013). This study was approved by the Biomedical Research Ethics Committee of Qujing First People’s Hospital of Kunming Medical University (202201), and the requirement for informed consent was waived.

Rectal cancer patients from January 2016 to December 2019 who underwent MR examination were retrospectively collected. The inclusion criteria were as follows: (1) patients who were pathologically diagnosed with rectal adenocarcinoma and (2) patients who underwent MR examination 1 week before surgery. The exclusion criteria were as follows: (1) MR images with motion artifact or metal artifact, (2) patients with other malignant tumors at the same time, (3) lack of pre-operative laboratory test results, (4) tumor deposits status was not tested. Finally, a total of 137 RC patients with TDs and 190 RC patients without TDs were enrolled in this study.

**Fig. 1:**
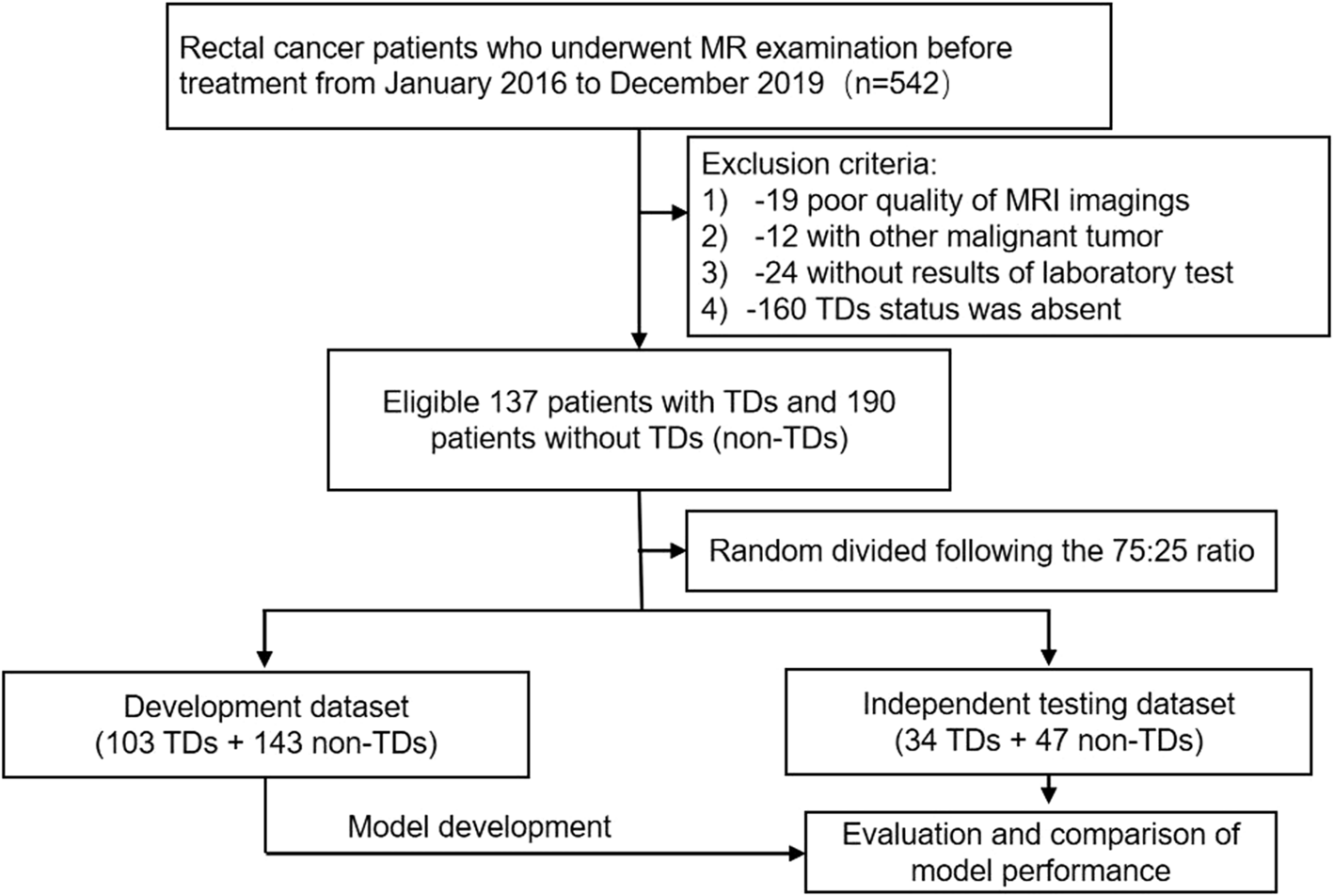
Flowchart of patient enrollment and study design

A pseudorandom number generator was applied to give a random number between 0 and 1 for each patient, and the patients with a random number less than 0.25 were selected as the testing dataset while the rest were used as the development dataset. Finally, the patients were divided into a development dataset (143 non-TDs and 103 TDs) and an independent testing set (47 non-TDs and 34 TDs). The predictive models were trained only in the development dataset, and the independent dataset was used as a held-out dataset for evaluating the model performance. The flowchart of patient enrollment is shown in Fig. 1.

### MRI examination protocol

All patients underwent MR examinations with 3.0-Tesla (T) scanners (MAGENTOM Skyra, Siemens Healthcare Sector, Germany; SIGNA Architect, GE Medical System, America; Ingenia Elition X, Philips, the Netherlands). Patients underwent some special bowel preparations before the MRI examination, including consuming liquid food on the day before the examination, and were administered a routine cleansing enema to ensure that the rectum was clear and empty 3 to 4 h before the examination. The main MR serial name was fast spin-echo no fat suppression T2-weighted imaging (FSE-T2WI). The parameters were set up as follows: repetition/echo time (TR/TE), 6890/100 ms; slice thickness, 3 mm; field of view (FOV), 220 × 220 mm; echo train length, 25; number of averages, 3; pacing between slices, 3.9 mm; flip angle, 150°; voxel size, 0.7 mm × 0.7 mm × 4.0 mm.

### MR image evaluation

Two professional radiologists (with 10 and 15 years of experience in RC diagnoses), who were blinded to patients’ information, reviewed the MR images. Lesions classified as T1 (the submucosa of the rectum was invaded) and T2 (the muscularis propria of the rectum was invaded) stages on MR images were integrated to form the MRI T1–2 cohorts, according to AJCC 8th edition of the TNM staging system. Nodules larger than 3 mm in diameter were considered positive lymph nodes on MR images. Apparent diffusion coefficient (ADC) value was measured with ADC mapping. CRM was considered positive if the distance between the mesorectal fascia and the outermost edge of the tumor was less than 1 mm. EMVI was considered positive if dense blood vessels were present around the tumor or vascular emptying signals were absent. The tumor location was measured based on the distance between the anal verge and lower pole of the tumor (bottom, ≤ 5 cm; middle, 5–10 cm; top, ≥ 10 cm). Differences of opinion were resolved through consultation during the image review. The baseline features of MR images of study patients are shown in Table 1.

**Table 1:** Comparison of patients’ characteristics in the development and testing datasets

Clinical variables	Development dataset			Independent testing dataset		
	Non-TDs (*n* = 143)	TDs (*n* = 103)	*p-value*	Non-TDs (*n* = 47)	TDs (*n* = 34)	*p-value*
Gender (female/male)	95/48	61/42	*0.247*	34/13	26/8	*0.676*
Age (mean ± SD)	57.6 ± 12.6	57.2 ± 12.3	*0.798*	59.0 ± 10.8	58.6 ± 12.3	*0.906*
MR T-stage (T1–2/T3/T4)	26/112/5	10/83/10	*0.034*	5/42/0	4/29/1	*0.486*
MR N-stage (N0/N1/N2)	75/53/15	14/60/29	< *0.001*	26/18/3	5/18/11	< *0.001*
MR M-stage (M0/M1)	137/6	99/4	*0.903*	47/0	33/1	*0.237*
Lesion location (bottom/mid/top/bottom-mid/mid-top/all)	2/13/59/14/53/2	25/16/17/19/23/3	< *0.001*	2/5/18/2/20/0	3/4/11/12/4/0	*0.001*
Morphologic type (mass/infiltrate)	24/119	34/69	*0.003*	8/39	16/18	*0.004*
CRM (− / +)	72/71	60/43	*0.220*	23/24	27/7	*0.005*
EMVI(− / +)	139/4	73/30	< *0.001*	46/1	22/12	< *0.001*
ADC value (mean ± SD)	0.914 ± 0.181	0.915 ± 0.136	*0.961*	0.905 ± 0.114	0.924 ± 0.135	*0.493*
CEA (− / +)	92/51	56/47	*0.115*	26/21	16/18	*0.463*
CA19-9 (− / +)	119/24	70/33	*0.005*	40/7	23/11	*0.062*
CA125 (− / +)	139/4	101/2	*0.668*	45/2	30/4	*0.203*

*Abbreviations: CRM* circumferential resection margin, *EMVI* extramural vascular invasion; CEA ( +), > 5 ng/m; CA19-9 ( +), > 35 u/ml; CA125 ( +), > 35 u/ml

### Pathological diagnosis of tumor deposits

Pathological reports recording pathological information of patients enrolled in this study were searched and downloaded from the Department of Pathology, West Chinese Hospital. The TD status (positive or negative) was described in the pathology reports, according to the standard of the AJCC 8th edition of the TNM staging system.

### Lesion segmentation

Two types of lesions (the tumor regions and the peri-tumoral regions) were manually annotated by two radiologists with 10 and 15 years of diagnostic experience on the MR images. Both the tumor regions and the peri-tumoral regions were manually labeled within the border of the ROI by using the ITK-SNAP software (v3.8.0, http://www.itksnap.org).

The peri-tumoral regions refer to the mesorectal fat surrounding the tumor, which fills the area between the tumor's edge and the mesorectal fascia. As recent studies [20, 21] had reported that peritumoral mesorectal fat played an important role in understanding the tumor microenvironment, predicting tumor recurrence, and assessing the aggressiveness and heterogeneity of rectal cancer, so we included peritumoral adipose tissue in analysis. We map the entire area between the tumor margin and the rectal fascia, instead of defining a 1- or 5-mm distance around the tumor, as the precise definition of a 1- or 5-mm distance was difficult and would increase the workload. An example of the manually segmented tumor region and corresponding peri-tumoral region is shown in Supplementary Fig. 1.

Both radiologists were blinded to the pathological reports. After the completion of the three-dimensional segmentation, 20 patients were randomly selected for evaluating feature stability. For the intra-class correlation analysis, radiologist A (with 10-year experience) drew ROI of those patients once again (1 month apart). For the inter-class correlation analysis, the correlation coefficient was calculated by comparing ROIs of radiologist A (first time) and radiologist B (with 15-year experience). The features with intra and inter-class correlation coefficient (ICC) ≤ 0.75 were excluded, according to commonly admitted rule that ICC > 0.75 = good or excellent reliability, < 0.5 = poor reliability, and 0.5–0.75 = moderate reliability [22].

### Data pretreatment

The clinical variables were recorded in relation to the TD diagnosis as follows:

(1) age, an actual variable; (2) gender, a dichotomous variable (female = 0, male = 1); (3) MR T stage, a polytomous variable (T1/T2 = 0, T3 = 1, T4 = 2); (4) MR N stage, a polytomous variable (N0 = 0, N1 = 1, N2 = 2); (5) MR M stage, a dichotomous variable (M0 = 0, M1 = 1); (6) lesion location, a polytomous variable (bottom = 0, middle = 1, top = 2, bottom/middle = 3, middle/top = 4, bottom/middle/top = 5); (7) tumor type, a dichotomous variable (mass = 0, infiltrative = 1); (8) CRM a dichotomous variable (negative = 0, positive = 1); (9) EMVI a dichotomous variable (negative = 0, positive = 1); (10) ADC value, an actual variable; (11) serum carcinoembryonic antigen (CEA) level, a dichotomous variable {negative = 0, positive = 1(> 5 ng/ml)}; (12) serum CA19-9 level, a dichotomous variable {negative = 0, positive = 1(> 35 u/ml)}; (13) serum carbohydrate antigen (CA)125 level, a dichotomous variable {negative = 0, positive = 1(> 35 u/ml)}.

Intensity normalization was first applied on the T2WI images to rescale the intensities to 0.255, and then the manually labeled ROIs on MR images were transformed and defined as follows before model development: (1) a three-dimensional (3D) patch containing the cropped ROI, which the size was determined on the largest ROI (64pixel × 64pixel × 10pixel for tumor regions, 128pixel × 128pixel × 10pixel for peri-tumoral regions); (2) manually labeled pixel-wise ROI masks, in which non-lesion areas were padded with zero; (3) the label of tumor deposits which was pathologically identified.

In order to avoid overfitting, data augmentation was applied during the training process of the deep learning models [23]. Flipping (at the *x* and *y* axis), random brightness contrast adjustment (80%, 90%, 110%, and 120%), and random rotation (90, 180, and 270° at the *z* axis) were performed. Finally, the sample size was increased to 2460 in the development dataset for the construction of the deep learning models.

### Development of the clinical model

To select the tumor deposits associated with clinical variables, both univariate regression analysis and multivariate regression analysis were carried out in the development dataset. The back stepwise selection was applied in multivariate regression analysis and only the clinical factors with a *p*-value less than 0.05 were selected for model development. A clinical model was constructed based on the selected clinical variables by applying logistic regression with the scikit-learn toolkit [24]. Liblinear was used as the default solver and an L2 regularization of C = 1 was also applied. The tolerance was set to 0.0001, other parameters were set by default.

### Design of the DL models

Based on whether integrating the peri-tumoral ROI images or clinical variables, three deep learning models were developed for our purpose: a single-channel deep learning (single-DL) model using only the tumor ROI as input, a multi-channel deep learning (multi-DL) model using both the tumor ROI image and peri-tumoral ROI image as input, and a hybrid deep learning (hybrid-DL) model that incorporating both tumor ROI and peri-tumoral ROI images as well as the selected clinical variables. For the single-DL model, ResNet50 was used as the backbone of the neural network for the extraction of high-dimension features from the tumor ROI images. The extracted image features were converted into a 1280-bit vector followed by a soft-max regression for the binary classification of TDs or non-TDs. For the multi-DL model, two ResNet50-based networks with fully connected layers were used for extracting high-dimension features from the ROIs containing tumor and peri-tumoral regions, respectively. These features were converted into 1280-bit vectors and further concatenated to 2560-bit vectors, and then the probability of TDs was predicted by the soft-max layer. The conceptual architecture of our single-DL and multi-DL models is shown in Supplementary Fig. 2.

**Fig. 2:**
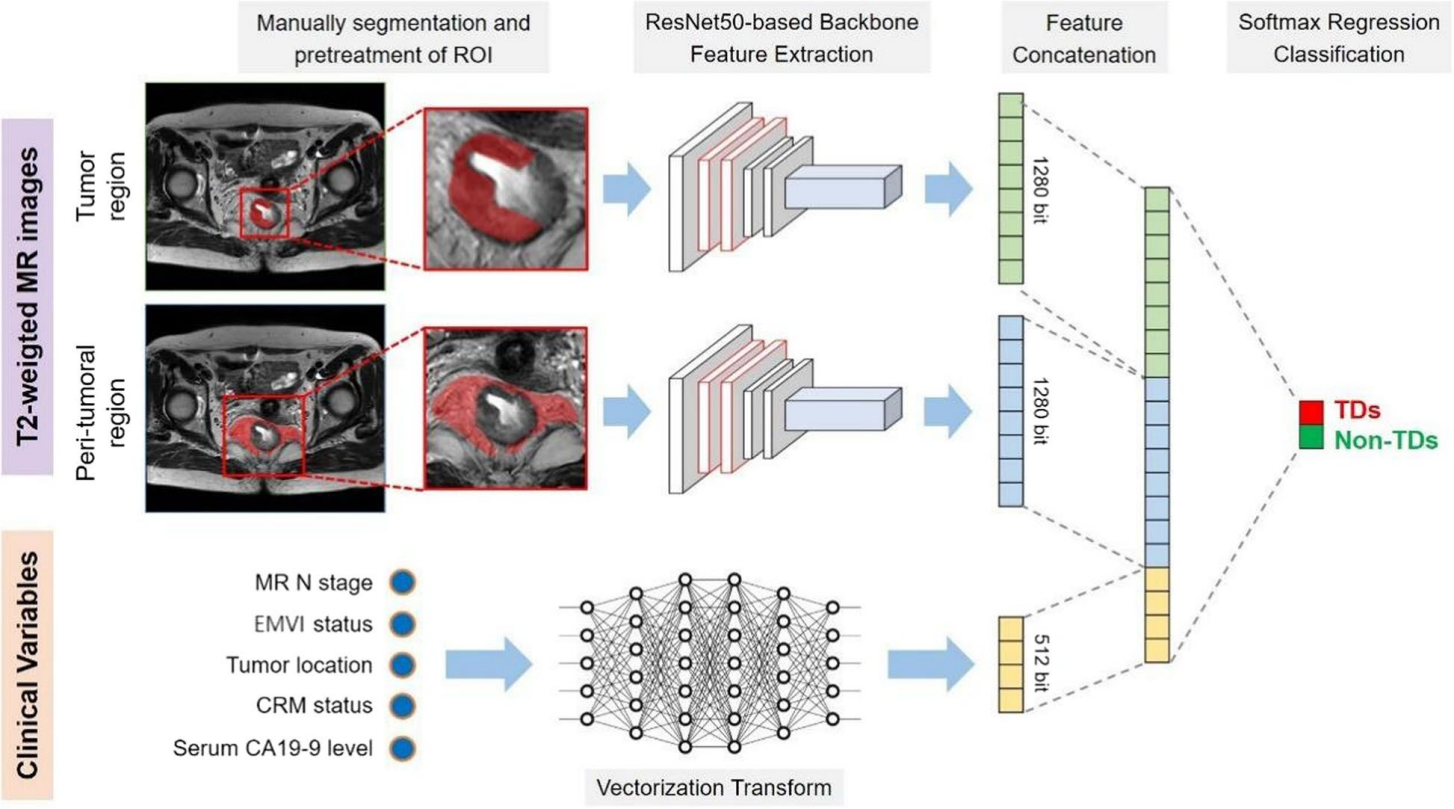
Conceptual architecture of the hybrid-DL model

For the hybrid-DL model, the selected clinical variables of each patient were first transformed into a 512-bit vector by using a fully connected layer and were concatenated with the transformed vectors from the tumor ROI as well as the peri-tumoral ROI. Finally, the concatenated 3072-bit vector integrating both MR image and clinical variables was used for assessing the probability of TDs in each patient (Fig. 2).

### Training of the DL models

To improve the adversarial robustness and transferability of the DL models, the transfer learning method was applied in this study. The neural networks were pretrained on the natural images from the ImageNet dataset and the medical images of multiple dataset from The Cancer Imaging Archive (TCIA) database [25, 26].

The weighted-balanced binary cross-entropy loss function was used to penalize errors of the majority class and value the minority class more positively, as class weights were inversely proportional to their frequency in the training data. The Mini-Batch Gradient Descent (MBGD) algorithm with a learning rate of 1e − 4 was used to optimize the parameters of the model and contribute to speeding up the convergence rate. The minibatch size and the dropout rate were set to 16 and 0.6, respectively. The other parameters were set as default. The training process was stopped when the loss in the validation set became stable, and the average number of epochs for the DL models varied from 60 to 80. The development of the proposed deep learning models was performed on the InferScholar platform (InferVision, China).

### Calibration and decision curve analysis

The calibration curve analysis was applied for the evaluation of consistency between the predicted TD probability and the actual observed rate, and the calibration curve was plotted by using 1000 bootstrapping resamples method. In addition, the goodness-of-fit of the predictive models was assessed with the Hosmer–Lemeshow test in the independent testing dataset [27]. Decision curve analysis (DCA) was also used to evaluate and compare the clinical usefulness of different models by calculating the net benefit across the reasonable range of threshold probabilities [28].

### Model interpretability

As the deep learning was “black-box” models, the Gradient-weighted Class Activation Mapping (Grad-CAM) approach was applied to better comprehend the DL models by highlighting the critical response areas on the T2WI images for the deep learning algorithms in predicting TDs [29]. For the single-DL and multi-DL models, the saliency maps were generated by applying Grad-CAM on the last convolutional layer of the deep learning models. In addition, beside the saliency map of the T2WI images, the relative weights of the selected clinical variables for each patient were also presented for the hybrid-DL model.

### Statistical analysis

All statistical analyses were performed with the MedCalc software (version 20.0) and the SPSS software (version 23.0). The continuous and the categorical variables were compared by the Mann–Whitney U test and the chi-square test, respectively. The discriminative capability of the predictive models was evaluated through the ROC analysis by assessing the AUC, and the sensitivity, specificity, positive predictive value (PPV), and negative predictive value (NPV) of each model were also calculated according to the optimal threshold which was determined by the maximum Youden index. The Delong’s test was used to compare the difference between two AUCs [30]. The calibration curve and the decision curve were generated with R language (version 3.6.4) by using the “rms” package and the “rmda” package, respectively. A two-sided *p* < 0.05 was considered statistically significant.

## Results

### Patient characteristics

The TD patients showed an improved MR N-stage, increased mass type and EMVI prevalence, and higher serum CA19-9 level than the non-TD patients in both development and independent testing datasets. There was no significant difference in the prevalence of TDs (*p* = 0.987) between the development dataset (42%, 103/246) and the independent testing dataset (42%, 34/81), as summarized in Table 1. There was no significant difference in gender, age, MR M stage, ADC value, serum CEA level, and serum CA125 level between the TD patients and non-TD patients in both development and independent testing datasets.

### Selection of the clinical variables

The clinical variables were analyzed before model development. After multivariate regression analysis, only the MR N-stage, EMVI, tumor location, CRM, and serum CA19-9 level showed a *p-*value less than 0.05 and were selected for model development (Table 2).

**Table 2:** Univariate and multivariate regression analysis of the clinical variables in the development dataset

Clinical variables	Univariate regression analysis			Multivariate regression analysis		
	Odds ratio	95% CI	*P*	Odds ratio	95% CI	*P*
Gender	1.363	0.807–2.302	*0.247*			
Age	0.997	0.975–1.019	*0.797*			
MR T-stage	2.172	1.177–4.006	*0.013*	1.497	0.687–3.261	*0.310*
MR N-stage	3.428	2.255–5.211	< *0.001*	3.549	2.153–5.852	< *0.001*
MR M-stage	0.923	0.254–3.356	*0.903*			
EMVI	14.281	4.845–42.098	< *0.001*	11.456	3.366–38.984	< *0.001*
Tumor location	0.687	0.565–0.835	< *0.001*	0.661	0.516–0.846	*0.001*
CRM	0.727	0.436–1.211	*0.041*	0.404	0.198–0.825	*0.013*
Morphologic type	0.409	0.224–0.746	*0.004*	0.707	0.335–1.493	*0.363*
ADC value	1.049	0.157–7.021	*0.960*			
Serum CEA level	1.514	0.903–2.539	*0.116*			
Serum CA125 level	0.6881	0.124–3.830	*0.670*			
Serum CA19-9 level	2.3375	1.279–4.272	*0.006*	2.356	1.139–4.874	*0.021*

*CRM* circumferential resection margin, *EMVI* extramural vascular invasion, *ADC* apparent diffusion coefficient

### Model performance comparison

Based on the manually segmented tumor ROI, peri-tumoral ROI, and selected clinical variables, the clinical model, single-DL model, multi-DL model, and hybrid DL model were constructed in the development dataset and evaluated in the independent testing dataset.

**Fig. 3:**
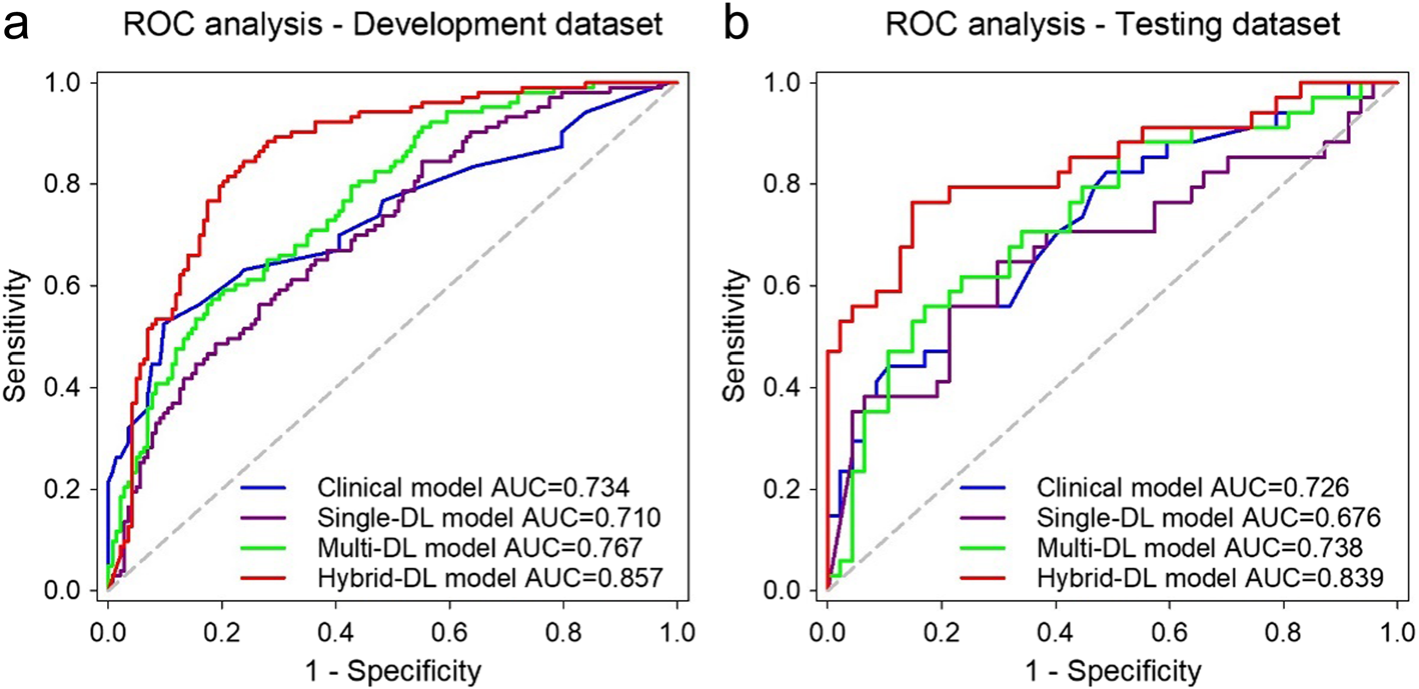
ROC analysis of the predictive models in the development (**a**) and testing (**b**) datasets

The diagnostic performance of these predictive models was compared through ROC analysis (Fig. 3). The clinical model showed AUCs of 0.734 (95% CI, 0.674–0.788) and 0.726 (95% CI, 0.615–0.819) in the development and the independent testing datasets, respectively. The single-DL model based on tumor ROI images had achieved AUCs of 0.710 (95% CI, 0.649–0.766) and 0.676 (95% CI, 0.563–0.776) in the development and the independent testing datasets, respectively. After incorporating peri-tumoral ROI images, the AUCs of the multi-DL model were improved to 0.767 (95% CI, 0.710–0.819) in the development dataset (*p* < 0.001) and 0.738 (95% CI, 0.628–0.829) in the independent testing dataset (*p* = 0.348). The results indicate that the peri-tumoral ROI could benefit TD diagnosis.

The hybrid-DL model showed favorable discrimination capability in the development dataset, with AUCs yielding 0.857 (95% CI, 0.807–0.898), and outperformed the clinical model (*p* = 0.068), single-DL model (*p* < 0.001), and multi-DL model (*p* < 0.001). Similar results were obtained in the independent testing dataset, although not statistically significant, the hybrid-DL model had achieved higher AUC (0.839 [95% CI, 0.741–0.911]) than that of the clinical model (*p* = 0.123), single-DL model *(p* = 0.028), and multi-DL model (*p* = 0.066). The sensitivity, specificity, PPV, and NPV of each model in the development and independent testing datasets are listed in Table 3.

**Table 3:** Performance comparison of the predictive models in the development and testing datasets

Dataset	Model	AUC (95% CI)	*p*-value	threshold	Sensitivity	Specificity	PPV	NPV
Development	Clinical	0.734 (0.674–0.788)	0.068	> 0.4487	52%	90%	79%	73%
	Single-DL	0.710 (0.649–0.766)	< 0.001	> 0.5433	56%	73%	60%	70%
	Multi-DL	0.767 (0.710–0.819)	< 0.001	> 0.4651	57%	82%	69%	73%
	Hybrid-DL	0.857 (0.807–0.898)	-	> 0.3086	85%	76%	72%	87%
Testing	Clinical	0.726 (0.615–0.819)	0.123	> 0.4280	56%	79%	66%	71%
	Single-DL	0.676 (0.563–0.776)	0.028	> 0.7583	65%	70%	61%	73%
	Multi-DL	0.738 (0.628–0.829)	0.066	> 0.4690	56%	83%	70%	72%
	Hybrid-DL	0.839 (0.741–0.911)	-	> 0.2210	77%	85%	79%	83%

### Clinical utility of the predictive models

**Fig. 4:**
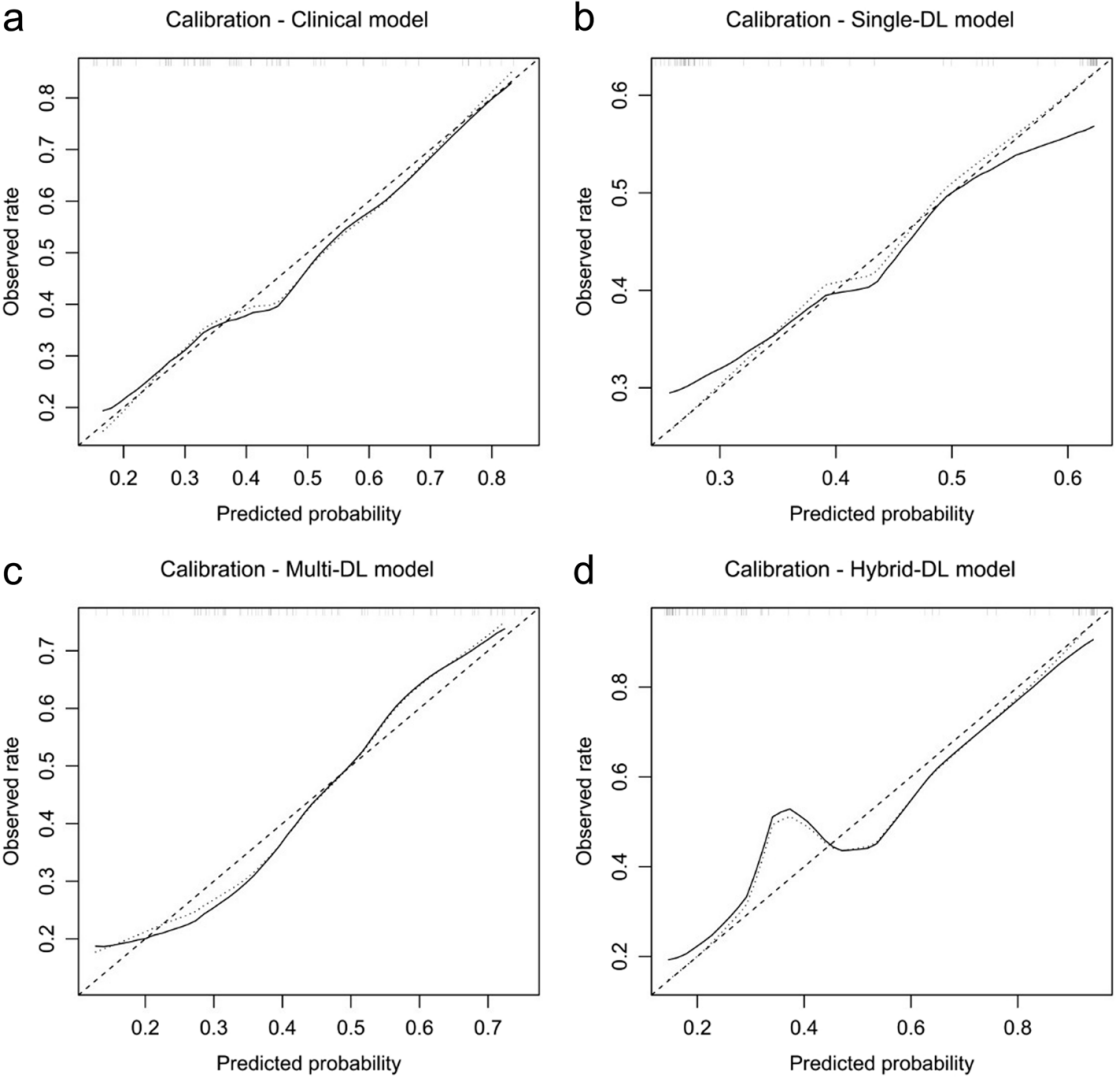
Calibration analysis of the clinical model (**a**), single-DL model (**b**), multi-DL model (**c**), and hybrid-DL model (**d**) in the testing dataset

All the predictive models showed good consistency between the actual observed rate and the predicted TD probability (Fig. 4). The Hosmer–Lemeshow test showed that the non-significant statistic was 0.919, 0.163, 0.941, and 0.092 for the clinical model, single-DL model, multi-DL model, and hybrid-DL model, respectively, which indicated no significant deviation of the models from an ideal fitting.

**Fig. 5:**
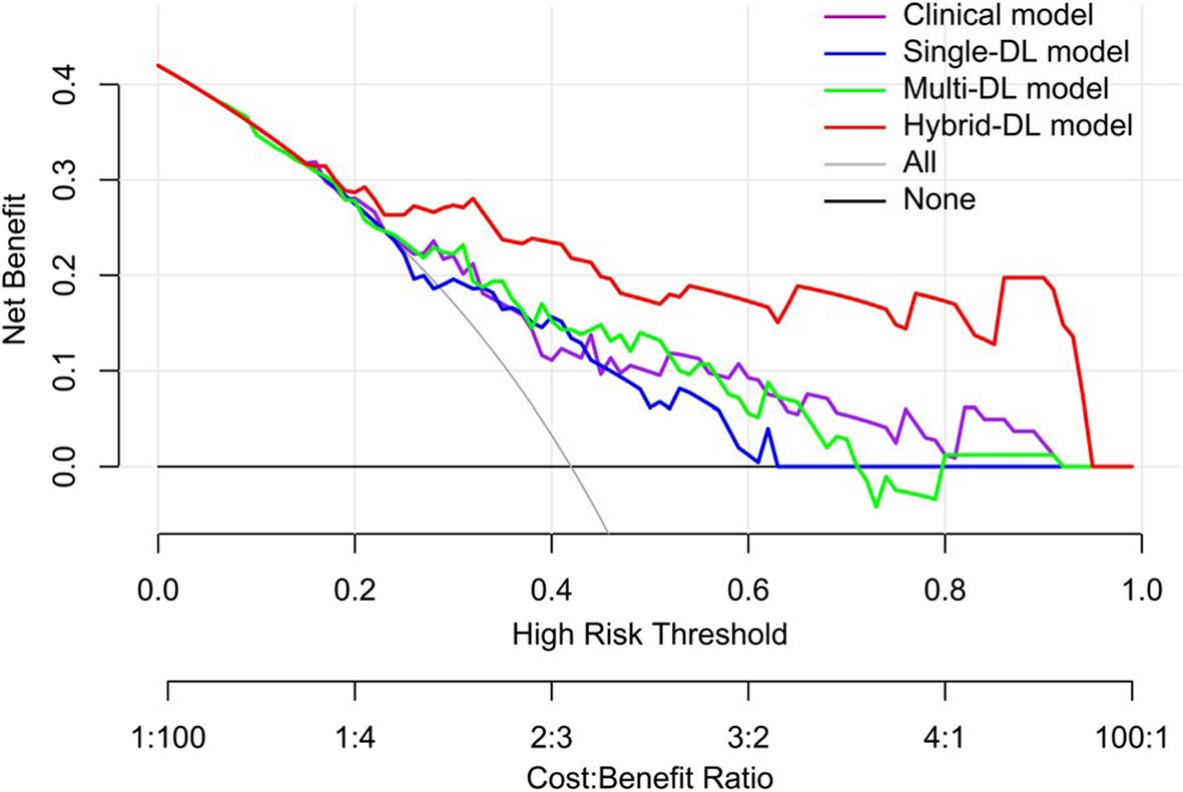
Decision curve analysis of the predictive models in the testing dataset

The DCA in the independent testing dataset demonstrated that the hybrid-DL model had higher net benefit than the other three models across almost the entire range of the threshold probabilities, which suggested that the hybrid-DL model was superior to other models in terms of clinical usefulness (Fig. 5).

### Interpretation of the DL models

**Fig. 6:**
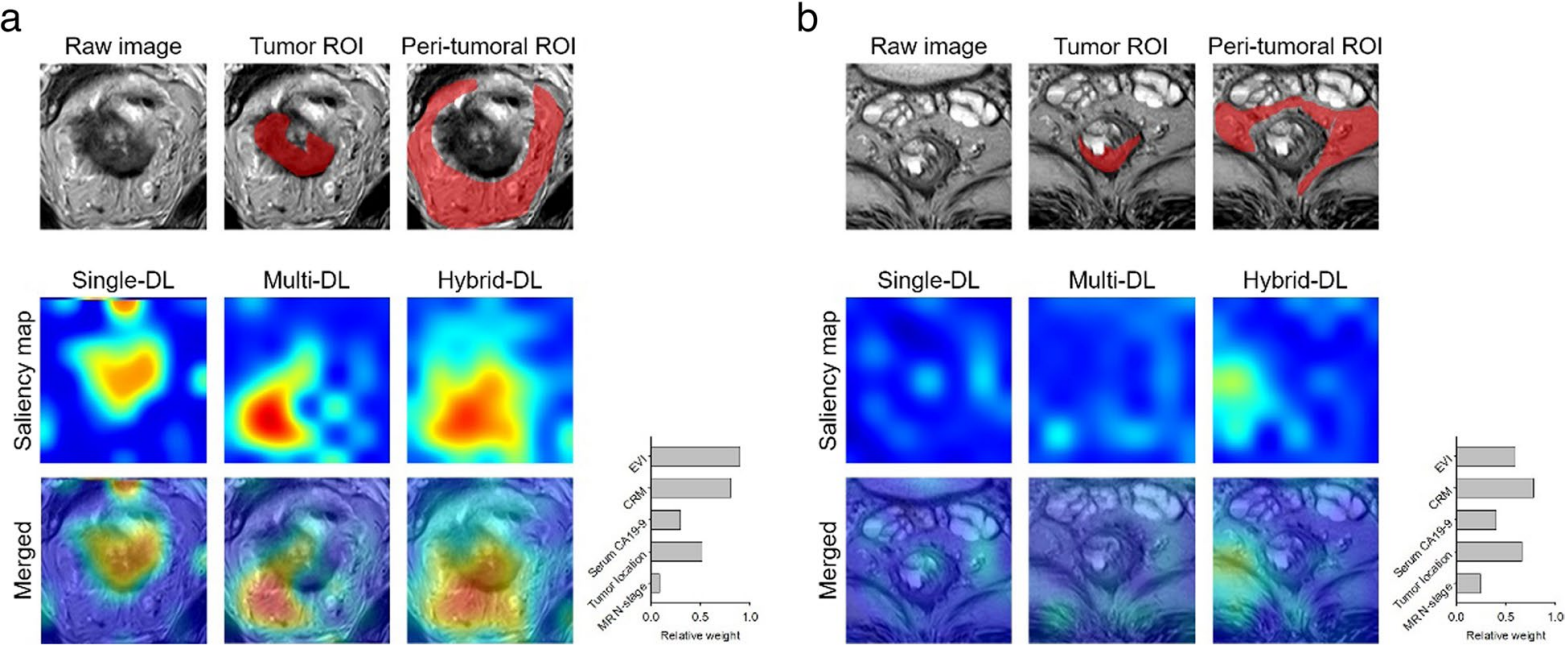
Saliency map analysis of the single-DL, multi-DL, and hybrid-DL models. **a** The saliency maps of a 67-year-old male patient with TDs. **b** The saliency maps of a 70-year-old female patient without TDs

The saliency maps of two representative cases are presented in Fig. 6. The distribution of the highlighted attentional regions was consistent with the manually labeled ROIs, and both tumor and peri-tumoral regions could contribute to TDs diagnosis in the multi-DL model and hybrid-DL model. In addition, the relative weights of the selected clinical variables changed among patients, indicating the personalized diagnosis mechanism of the Hybrid-DL model.

## Discussion

In the present study, we developed three DL models and a clinical model to predict TDs in RC patients. In the independent testing dataset, the hybrid DL model showed superior diagnostic capability compared to other models (AUC = 0.839; sensitivity = 77%; specificity = 85%). To our knowledge, this is the first study assessing the feasibility of DL on MR imaging to predict TDs in RC patients preoperatively.

As an important prognostic factor in RC, the presence of TDs is associated with perineural, lymphatic, or vascular invasion [31]. More importantly, TDs are correlated with the N stage of the tumor and have implications for the selection of treatment strategies, e.g., Nagtegaal et al. [32] demonstrated that patients with TDs had the worst outcome whether there were LNMs or not. Goldstein et al. also reported that the patients with TDs always demonstrated a worse survival when their numbers of metastatic LNs differed [33]. However, a major obstacle to TD classification is that no reliable diagnostic method of TDs on the traditional imaging by gross eye has been established [34]. Hence, an objective and accurate assessment method for predicting TDs in RC patients before surgery is urgently required and of great clinical significance. If we can predict the presence of TDs preoperatively, we can avoid overestimating patients’ prognoses and refraining from forgoing nCRT, which has the potential to significantly enhance patient outcomes, and improve patients’ survival rates. Although identification of TDs in RC patients through radiomics analysis on MR, CT, and US images had been reported in previous literature [35, 36], radiomic feature quantification was sensitive to acquisition modes and matrix sizes, and the delineation of tumor volumes was also crucial for the assessment of radiomics features [37]. On the other hand, many radiomics features were found to be unstable between two scans acquired within weeks or even 15 min of each other [38]. As a special type of artificial neural network, deep learning can automatically extract high-level features directly from images and has achieved impressive success in medical analysis for its image-based pattern recognition [39]. Compared with previous radiomics analysis, several advantages of the DL model in this study could be highlighted. First, our study has enrolled more patients than previous studies (327 vs 40 ~ 254) and the larger sample size could lead to lower bias and better model reliability, which is confirmed by the similar performance of the hybrid-DL model between development and independent testing datasets. Second, more risk factors including clinical characteristics, MR findings, and serum biomarkers were used in our study, which could provide more valuable information and improve model performance. Third, our DL models are trained end-to-end supervised method, avoiding the limitation of pre-defined hand-crafted radiomics features [40].

Interestingly, in this study, we found that the incorporation of peri-tumoral fat regions could benefit DL-based TD diagnosis, as the multi-DL model achieved higher AUC than that of the single-DL model in both development and independent testing datasets. The potential mechanisms may be that TDs via neurotrophic extravascular migratory and perineural invasion, which could lead to radiographic changes around the tumor regions [41, 42], so the analysis of peritumoral adipose tissue could contribute to the detection of TDs. Previous studies [20, 21] had reported that peritumoral mesorectal fat played an important role in understanding the tumor microenvironment, predicting tumor recurrence, assessing the aggressiveness and heterogeneity of rectal cancer, and our finding was consistent with previous studies.

It should be noted that there were several limitations in this study. Firstly, as a single-center retrospective analysis, only RC patients confirmed by surgical pathology were enrolled in this study, and selection bias might be unavoidable, and the generalizability of our findings to a broader patient population may be compromised. To address this limitation, a multicenter study with more enrolled patients should be conducted to enhance the robustness and external validity of our conclusions. Secondly, the DL model was built on T2-weighted MR images. Multi-MR sequences included in the building of the model may potentially improve the reliability and applicability of the model to other different image datasets. Therefore, model incorporating other MR sequences such as diffusion-weighted imaging need to be investigated in the future. Third, the inability to automatically delineate the target region and issues may weaken the reproducibility of the model, and a method that can automatically delineate the target region and issues needs to be used in the future.

## Conclusions

The presence of tumor deposits has been found to be associated with both the tumor stage and the treatment strategy in patients with rectal cancer. The non-invasive identification of TDs holds significant clinical implications. In this study, we developed four distinct models, comparing their diagnostic performance with the aim of furnishing a valuable predictive tool for TD status. Furthermore, our investigation revealed that the model utilizing deep learning techniques exhibited superior performance compared to either a single radiomics or clinical model. Specifically, hybrid DL model integrating T2-MR images and clinical risk factors demonstrated robust predictive capabilities and holds promise as an asset in predicting TDs in rectal cancer cases, and the application of deep learning methodologies in clinical settings exhibits promising prospects.

### Supplementary Information

**Additional file 1: Supplementary Figure 1.** Example of the manual three-dimensional segmentation results. **Supplementary Figure 2.** Workflow chart and schematic of the single-DL model (A) and the multi-DL model (B).

## Data Availability

This is a free-access article. Supplementary Material and the primary article both contain the original data.

## Abbreviations

ADC: Apparent diffusion coefficient
AJCC: American Joint Committee on Cancer
AUC: Area under the curve
CA19-9: Carbohydrate antigen19-9
CEA: Carcinoembryonic antigen
CNN: Convolutional neural network
CRC: Colorectal cancer
CRM: Circumferential resection margin
DCA: Decision curve analysis
DL: Deep learning
EMVI: Extramural vascular invasion
FOV: Field of view
FSE-T2WI: Fast spin-echo no fat suppression T2-weighted imaging
IARC: International Agency for Research on Cancer
LNM: Lymph node metastasis
NPV: Negative predictive value
PPV: Positive predictive value
ROC: Receiver operating characteristics
ROI: Regions of interest
T2WI-MRI: T2-weighted magnetic resonance imaging
TCIA: Cancer Imaging Archive
TDs: Tumor deposits
TR/TE: Repetition/echo time

## References

[1] Mattiuzzi C, Sanchis-Gomar F, Lippi G (2019) Concise update on colorectal cancer epidemiology. Ann Transl Med 7:609. 10.21037/atm.2019.07.91 10.21037/atm.2019.07.91 PMC7011596 32047770

[2] Morgan E, Arnold M, Gini A (2023). Global burden of colorectal cancer in 2020 and 2040: incidence and mortality estimates from globocan. Gut.

[3] Li M, Xu G, Chen Q, et al (2023) Computed tomography-based radiomics nomogram for the preoperative prediction of tumor deposits and clinical outcomes in colon cancer: a multicenter study. Acad Radiol 1572–1583. 10.1016/j.acra.2022.11.005 10.1016/j.acra.2022.11.005 36566155

[4] Lord AC, Graham Martínez C, D’Souza N (2019). The significance of tumour deposits in rectal cancer after neoadjuvant therapy: a systematic review and meta-analysis. Eur J Cancer.

[5] Wang Y, Zhang J, Zhou M, et al (2019) Poor prognostic and staging value of tumor deposit in locally advanced rectal cancer with neoadjuvant chemoradiotherapy. Cancer Med 8:1508–1520. 10.1002/cam4.2034 10.1002/cam4.2034 PMC6488131 30790459

[6] Fata CR, Gonzalez RS, Liu E (2017). Mesenteric tumor deposits in midgut small intestinal neuroendocrine tumors are a stronger indicator than lymph node metastasis for liver metastasis and poor prognosis. Am J Surg Pathol.

[7] Glynne-Jones R, Wyrwicz L, Tiret E, et al (2018) Rectal cancer: ESMO Clinical Practice Guidelines for diagnosis, treatment and follow-up. Ann Oncol 29:263. 10.1093/annonc/mdy161. 10.1093/annonc/mdy161 29741565

[8] Schaap DP, Voogt ELK, Burger JWA (2021). Prognostic implications of MRI-detected EMVI and tumor deposits and their response to neoadjuvant therapy in cT3 and cT4 rectal cancer. Int J Radiat Oncol Biol Phys.

[9] Zhu HT, Zhang XY, Shi YJ (2022). The conversion of MRI data with multiple b-values into signature like pictures to predict treatment response for rectal cancer. J Magn Reson Imaging.

[10] Yang YS, Feng F, Qiu YJ, Zheng GH, Ge YQ, Wang YT (2021) High-resolution MRI-based radiomics analysis to predict lymph node metastasis and tumor deposits respectively in rectal cancer. Abdom Radiol (NY) 46:873–884. 10.1007/s00261-020-02733-x 10.1007/s00261-020-02733-x 32940755

[11] Atre ID, Eurboonyanun K, Noda Y, et al (2021) Utility of texture analysis on T2-weighted MR for differentiating tumor deposits from mesorectal nodes in rectal cancer patients, in a retrospective cohort. Abdom Radiol (NY) 46:459–468. 10.1007/s00261-020-02653-w 10.1007/s00261-020-02653-w 32700214

[12] Pavic M, Bogowicz M, Würms X, et al (2018) Influence of inter-observer delineation variability on radiomics stability in different tumor sites. Acta Oncol 57:1070–1074. 10.1080/0284186X.2018.1445283 10.1080/0284186X.2018.1445283 29513054

[13] Yang F, Dogan N, Stoyanova R, et al (2018) Evaluation of radiomic texture feature error due to MRI acquisition and reconstruction: a simulation study utilizing ground truth. Phys Med 50:26–36. 10.1016/j.ejmp.2018.05.017 10.1016/j.ejmp.2018.05.017 29891091

[14] Xu J, Zhang R, Zhou Z, et al (2021) Deep network for the automatic segmentation and quantification of intracranial hemorrhage on CT. Front Neurosci 14:541817. 10.3389/fnins.2020.541817 10.3389/fnins.2020.541817 PMC7832216 33505231

[15] McKinney SM, Sieniek M, Godbole V, et al (2020) International evaluation of an AI system for breast cancer screening. Nature 586:E19. 10.1038/s41586-020-2679-9 10.1038/s41586-020-2679-9 33057216

[16] Xu Y, Hosny A, Zeleznik R, et al (2019) Deep learning predicts lung cancer treatment response from serial medical imaging. Clin Cancer Res 25:3266–3275. 10.1158/1078-0432.CCR-18-2495. 10.1158/1078-0432.CCR-18-2495 PMC6548658 31010833

[17] Coudray N, Ocampo PS, Sakellaropoulos T, et al (2018) Classification and mutation prediction from non–small cell lung cancer histopathology images using deep learning. Nat Med 24:1559–1567. 10.1038/s41591-018-0177-5 10.1038/s41591-018-0177-5 PMC9847512 30224757

[18] Zhang W, Huang Z, Zhao J, et al (2021) Development and validation of magnetic resonance imaging-based radiomics models for preoperative prediction of microsatellite instability in rectal cancer. Ann Transl Med 9:134. 10.21037/atm-20-7673 10.21037/atm-20-7673 PMC7867944 33569436

[19] Jang BS, Lim YJ, Song C, et al (2021) Image-based deep learning model for predicting pathological response in rectal cancer using post-chemoradiotherapy magnetic resonance imaging. Radiother Oncol 161:183–190. 10.1016/j.radonc.2021.06.019 10.1016/j.radonc.2021.06.019 34139211

[20] Pan AF, Zheng NX, Wang J, et al (2022) Role of perirectal fat in the carcinogenesis and development of early-onset rectal cancer. J Oncol 2022:4061142. 10.1155/2022/4061142 10.1155/2022/4061142 PMC8965599 35368890

[21] Felsenreich DM, Gachabayov M, Bergamaschi R (2023). Does the mesorectal fat area impact the histopathology metrics of the specimen in males undergoing tme for distal rectal cancer?. Updates Surg.

[22] Benchoufi M, Matzner-Lober E, Molinari N, Jannot AS, Soyer P (2020). Interobserver agreement issues in radiology. Diagn Interv Imaging.

[23] Shorten C, Khoshgoftaar TM (2021) A survey on image data augmentation for deep learning. J Big Data 55:2351–2377. 10.1007/s10462-021-10066-4 10.1186/s40537-021-00492-0 PMC8287113 34306963

[24] Bac J, Mirkes EM, Gorban AN, Tykin I, Zinovyev A (2011) Scikit-learn: machine learning in Python. Entropy. 10.3390/e23101368 10.3390/e23101368 PMC8534554 34682092

[25] Russakovsky O, Deng J, Su H, et al (2015) ImageNet large scale visual recognition challenge. Int J Comput Vis 37:1904–16. 10.1109/TPAMI.2015.2389824

[26] Clark K, Vendt B, Smith K, et al (2013) The cancer imaging archive (TCIA): Maintaining and operating a public information repository. J Digit Imaging 26:1045–57. 10.1007/s10278-013-9622-7 10.1007/s10278-013-9622-7 PMC3824915 23884657

[27] Finazzi S, Poole D, Luciani D, et al (2011) Calibration belt for quality-of-care assessment based on dichotomous outcomes. PLoS One 6:e16110. 10.1371/journal.pone.0016110 10.1371/journal.pone.0016110 PMC3043050 21373178

[28] Vickers AJ, Elkin EB (2006) Decision curve analysis: a novel method for evaluating prediction models. Med Decis Making 26:565–74. 10.1177/0272989X06295361 10.1177/0272989X06295361 PMC2577036 17099194

[29] Selvaraju RR, Cogswell M, Das A, et al (2022) Grad-CAM: visual explanations from deep networks via gradient-based localization. Int J Comput Vis 132:102382. 10.1016/j.artmed.2022.102382

[30] DeLong ER, DeLong DM, Clarke-Pearson DL (1988) Comparing the areas under two or more correlated receiver operating characteristic curves: a nonparametric approach. Biometrics 44:837–45. 3203132 3203132

[31] Kim S, Huh JW, Lee WY (2023). Prognostic impact of lymphatic invasion, venous invasion, perineural invasion and tumor budding in rectal cancer treated with neoadjuvant chemoradiotherapy followed by total mesorectal excision. Dis Colon Rectum.

[32] Nagtegaal ID, Knijn N, Hugen N, et al (2017) Tumor deposits in colorectal cancer: Improving the value of modern staging-a systematic review and meta-analysis. J Clin Oncol 35:1119–1127. 10.1200/JCO.2016.68.9091 10.1200/JCO.2016.68.9091 28029327

[33] Goldstein NS, Turner JR (2000) Pericolonic tumor deposits in patients with T3N+M0 colon adenocarcinomas: markers of reduced disease free survival and intra-abdominal metastases and their implications for TNM classification. Cancer 88:2228–38. 10820343 10820343

[34] Chen L Da, Li W, Xian MF, et al (2020) Preoperative prediction of tumour deposits in rectal cancer by an artificial neural network–based US radiomics model. Eur Radiol 30:1969–1979. 10.1007/s00330-019-06558-1 10.1007/s00330-019-06558-1 31828415

[35] Jin Y, Li M, Zhao Y, et al (2021) Computed tomography-based radiomics for preoperative prediction of tumor deposits in rectal cancer. Front Oncol 11:710248. 10.3389/fonc.2021.710248 10.3389/fonc.2021.710248 PMC8502898 34646765

[36] Ibrahim A, Primakov S, Beuque M, et al (2021) Radiomics for precision medicine: Current challenges, future prospects, and the proposal of a new framework. Methods 188:20–29. 10.1016/j.ymeth.2020.05.022 10.1016/j.ymeth.2020.05.022 32504782

[37] Galavis PE, Hollensen C, Jallow N,et al (2010) Variability of textural features in FDG PET images due to different acquisition modes and reconstruction parameters. Acta Oncol 49:1012–6. 10.3109/0284186X.2010.498437 10.3109/0284186X.2010.498437 PMC4091820 20831489

[38] Balagurunathan Y, Kumar V, Gu Y, et al (2014) Test–retest reproducibility analysis of lung CT image features. J Digit Imaging 27:805–23. 10.1007/s10278-014-9716-x 10.1007/s10278-014-9716-x PMC4391075 24990346

[39] Zhou SK, Greenspan H, Davatzikos C (2021). A review of deep learning in medical imaging: imaging traits, technology trends, case studies with progress highlights and future promises. PIEEE.

[40] Sun Q, Lin X, Zhao Y, et al (2020) Deep learning vs. radiomics for predicting axillary lymph node metastasis of breast cancer using ultrasound images: don’t forget the peritumoral region. Front Oncol 10:53. 10.3389/fonc.2020.00053 10.3389/fonc.2020.00053 PMC7006026 32083007

[41] Lee HS, Lee HE, Yang HK, et al (2013) Perigastric tumor deposits in primary gastric cancer: Implications for patient prognosis and staging. Ann Surg Oncol 20:1604–13. 10.1245/s10434-012-2692-9 10.1245/s10434-012-2692-9 23184289

[42] Lugassy C, Zadran S, Bentolila LA, et al (2014) Angiotropism, pericytic mimicry and extravascular migratory metastasis in melanoma: an alternative to intravascular cancer dissemination. Cancer Microenviron 7:139–52. 10.1007/s12307-014-0156-4 10.1007/s12307-014-0156-4 PMC4275501 25304454

