# Supplementary material for: Predicting tumor deposits in rectal cancer: a combined deep learning model using T2-MR imaging and clinical features
Source: Insights Imaging. 2023 Dec 20;14:221. doi: 10.1186/s13244-023-01564-w (PMC10733230; doi:10.1186/s13244-023-01564-w)
Supplement: Supplementary file 1 — Additional file 1: Supplementary Figure 1. Example of the manual three-dimensional segmentation results. Supplementary Figure 2. Workflow chart and schematic of the single-DL model (A) and the multi-DL model (B). [file 13244_2023_1564_MOESM1_ESM.docx]

**Predicting tumor deposits in rectal cancer: A combined deep learning model using T2-MR imaging and clinical features**

**ELECTRONIC SUPPLEMENTARY MATERIAL**

**
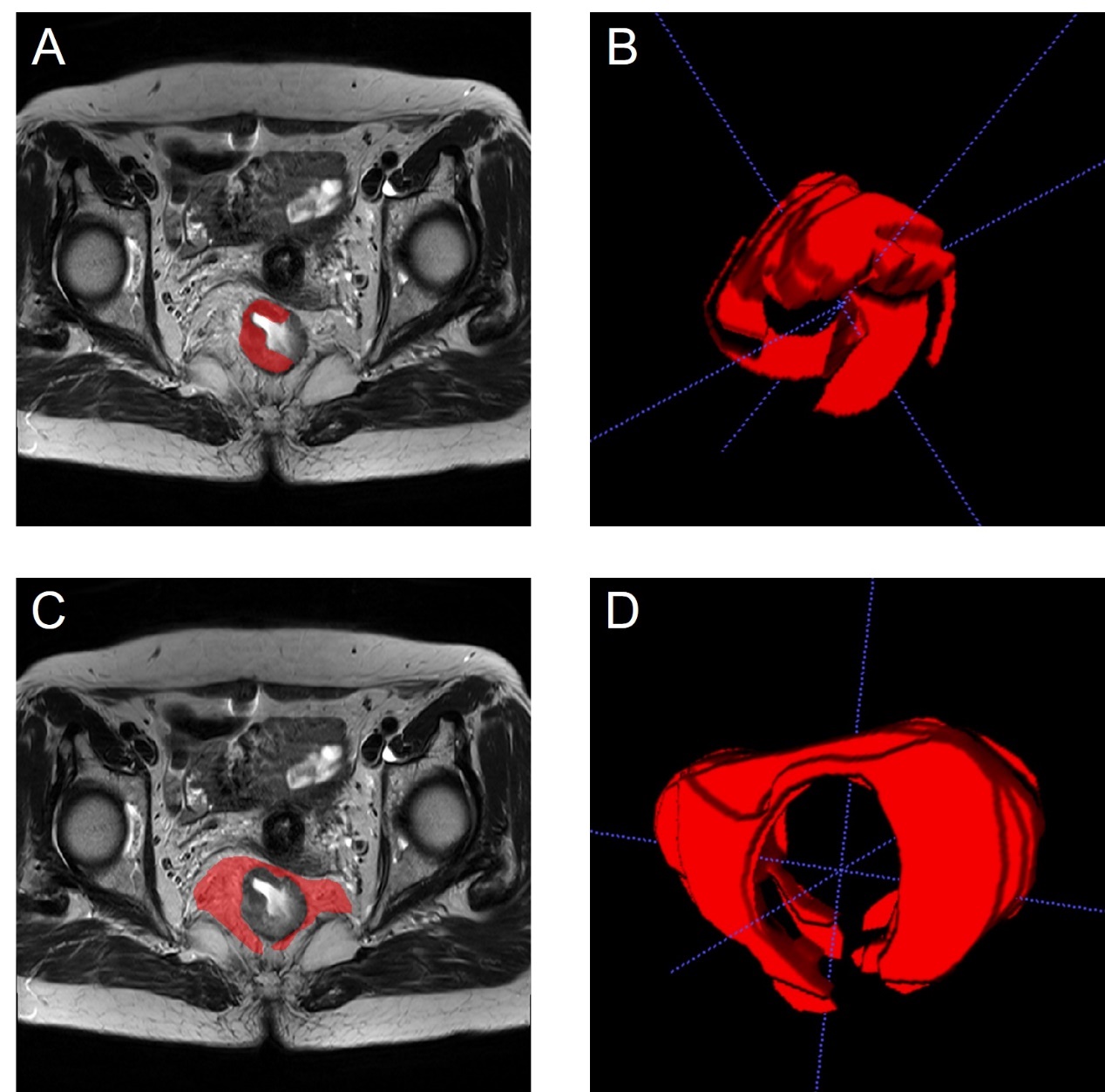
**

**Supplementary Figure 1.** Example of the manual three-dimensional segmentation results. (A) Segmentation of the tumor region on the axial slice. (B) Three-dimensional volumetric reconstruction of the segmented tumor region. (C) Segmentation of the peri-tumoral region on the axial slice. (D) Three-dimensional volumetric reconstruction of the segmented peri-tumoral region.


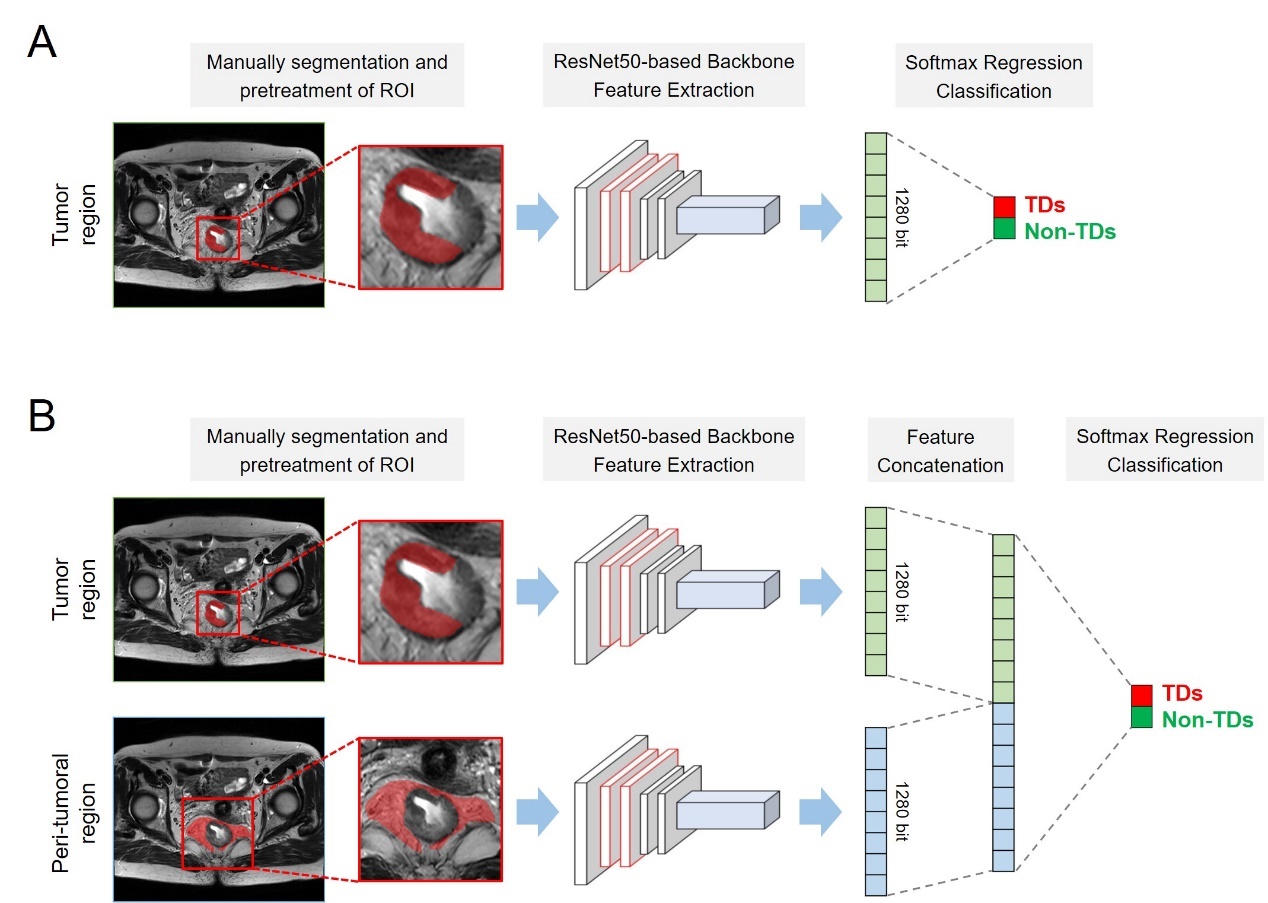


**Supplementary Figure 2.** Workflow chart and schematic of the single-DL model (A) and the multi-DL model (B)
